# Quadrant classification and development path of gymnastics-related research topics in Chinese high-quality journals: Based on SCI, PKU Core and CSSCI source journals

**DOI:** 10.1371/journal.pone.0355670

**Published:** 2026-11-02

**Authors:** Jingli Yu, Wen Sun, Luping Qu, Min Wang, JiaJia Zhang, Chenyao Zhang

**Affiliations:** 1 School of Sports Science, Qufu Normal University, Qufu, Shandong, China; 2 School of Physical Education, Huzhou University, Huzhou, Zhejiang, China; Dalian Polytechnic University, CHINA

## Abstract

Against the macro backdrop of the strategy for developing a strong sporting nation and digital-intelligent transformation, sports organizations have accumulated massive high-value unstructured knowledge assets such as academic literature and reports. Yet their latent strategic value has not been effectively exploited. Converting static knowledge assets into dynamic strategic intelligence represents the central challenge in sports knowledge management.Taking 169 high quality journal papers on gymnastics published from 2016 to 2025 in the China National Knowledge Infrastructure (CNKI, domestic edition) as research objects, this study identifies seven Topics using the Latent Dirichlet Allocation (LDA) model: Topic 1: Technical analysis of bar transitions and choreography in gymnastics competitions; Topic 2: Evolution of the global team structure in men’s artistic gymnastics; Topic 3: Biomechanical analysis of landing techniques among gymnasts; Topic 4: Research on curriculum design and instructional practice in school gymnastics education; Topic 5: Research on balance capability in ‘gymnasts’ apparatus performances; Topic 6: Movement technique analysis of elite gymnasts; Topic 7: Talent cultivation for reserve forces in competitive gymnastics. Quantitative Topic evaluation shows that Topic 4 is a high-concentration and high-coverage Topic; Topic 5 and 6 are high-concentration and low-coverage Topics; Topic 1 is a low-concentration and low-coverage Topic; and Topic 2, 3, and 7 are low-concentration and high-coverage Topics. This study conducts a Topic-based four-quadrant classification diagnosis and proposes differentiated development paths. Accordingly, it identifies future research directions for Topics in each quadrant, including theoretical deepening, application expansion, basic cultivation, and knowledge integration.

## 1. Introduction

Gymnastics-related sports constitute a multidisciplinary domain encompassing sports training, injury prevention, biomechanics, exercise physiology and sports psychology. As a traditional advantageous event for China in the Olympic Games and other major competitions, gymnastics has long maintained competitiveness in international tournaments. In recent years, the volume of academic literature in relevant fields has increased substantially, which creates an urgent need for systematic, data-driven approaches to map its knowledge structure and research Topics. A review of existing studies shows that although bibliometric and topic-modeling investigations in the field of sports have gradually been carried out, systematic quantitative research targeting the multidisciplinary interdisciplinary domain of gymnastics remains relatively limited. Current studies are characterized by fragmented research objects and the absence of comprehensive evaluations; their methodological paradigms are dominated by descriptive bibliometrics with insufficient application of topic modeling. Čolakovac et al. conducted a bibliometric analysis of artistic gymnastics, yet their work focused merely on a single modality and relied primarily on descriptive statistics, without topic modeling or multi-indicator comprehensive evaluation [1]; Rodriguez Redondo and Leon et al. conducted a bibliometric analysis of scientific outputs on the male and female modalities of artistic gymnastics in Scopus and Web of Science. They identified a total of 482 publications covering the period from 1975 to 2023, and summarized four major research lines: technical analysis, physiological aspects, gymnastics as a foundational sport, as well as sports evaluation and scoring [2]; Vargas and Capraro, in contrast, specifically examined the academic output of men’s artistic gymnastics, identifying 52 publications spanning the period from 1994 to 2019 [3]. These studies have laid a solid bibliometric foundation for artistic gymnastics, yet their research scope is confined exclusively to artistic gymnastics. In view of this, a review of existing studies reveals that systematic bibliometric investigations in the field of gymnastics are relatively limited. Relevant research mainly focuses on publication metrics and citation-network analysis of the single modality of artistic gymnastics. Few studies have conducted comprehensive quantitative evaluations that integrate competitive gymnastics, artistic gymnastics and aerobic gymnastics within a unified analytical framework. In recent years, the LDA topic model and multi-indicator comprehensive evaluation method have been gradually introduced into bibliometric research of sports [4,5], providing a computable empirical path for the identification of potential research Topics. This study adopts the entropy-weight method to implement objective comprehensive weighting for AIC, BIC, log-likelihood and JSD. Based on the dual logic of statistical goodness-of-fit and topic distinctiveness, this paper takes high-quality gymnastics-related research papers published in the CNKI database from 2016 to 2025 as research objects. It aims to bring competitive gymnastics, artistic gymnastics and aerobic gymnastics into the same bibliometric analytical framework. Furthermore, on the basis of four-quadrant classification diagnosis, differentiated development paths are deduced, so as to offer empirical references for the systematic development and knowledge optimization of gymnastics scientific research.

### 1.1. Value examination of gymnastics-related research papers in Chinese high-quality journals

Although Chinese gymnastics occupies a prominent position in international competitions, it still faces numerous practical challenges in the new historical development stage. To explore solutions to these problems, a systematic review of existing domestic gymnastics research outcomes is urgently required. Based on high-quality journal papers, this chapter discusses the core values of gymnastics research, laying a theoretical foundation for subsequent topic mining and strategy formulation.

#### 1.1.1. Gymnastics-related research literature constitutes the theoretical foundation for the scientific development of the gymnastics discipline.

The Opinions on Promoting the Prosperity and Development of Academic Journals, issued by the Publicity Department of the Communist Party of China Central Committee, the Ministry of Education, and the Ministry of Science and Technology, states that “academic journals are important platforms for academic research and exchange, key fronts for disseminating ideas and culture, and vital forces for advancing theoretical innovation and scientific and technological progress. Strengthening the development of academic journals plays a significant role in enhancing national scientific and technological competitiveness and cultural soft power” [6]. High-quality journals adhere to stringent review criteria and represent one of the most important carriers of high academic value in disciplinary research, characterized by authority and leadership. Research findings published in such journals that feature theoretical breakthroughs and methodological innovations not only reflect individual scholars’ academic competence but also epitomize the developmental level of a discipline, recognition from the academic community, and the capacity for translating research outcomes. In biomechanical research, studies on inertial sensor-based gymnastics motion capture and simulation technologies enable accurate measurement and visualization of movements [7], furthermore, by precisely simulating contact and constraint relationships between body segments within computer models, such approaches enhance the biomechanical authenticity and predictive capability of simulations for complex human movements [8]. In cognitive psychology research, investigations focusing on the cognition-emotion interaction of adolescent athletes reveal the differentiated demands that various sport types impose on psychological capacities [9]. In training science research, studies on the scientific arrangement of auxiliary training frequency and the specific relationship between load and adaptation transform scientific training into a precise operational process based on quantitative monitoring [10,11], goal orientation, and dynamic adjustment. This thereby enhances training efficiency, reduces injury risks, and maximizes athletes’ competitive performance.

#### 1.1.2. Gymnastics research literature serves as a practical guide for the high-quality development of the gymnastics cause.

Gymnastics research published in high quality journals plays a theoretically guiding role between policy and practice. It constitutes vital theoretical support for promoting the scientization of gymnastics training and the rationalization of rule-setting, thereby advancing the high-quality development of gymnastics across three dimensions: theoretical innovation, practical guidance, and policy translation. The study reveals that the shortage of reserve talents in competitive gymnastics has become a bottleneck restricting the development of gymnastics in China [12,13], to tackle the problem of talent discontinuity in reserve forces, relevant literature uses case studies to establish a cultivation model under the perspective of “sports-education integration.” It underscores scientific training and diversified management, providing replicable operational frameworks for grassroots training organizations [14]. Furthermore, in December 2024, the Gymnastics Center of the General Administration of Sport of China issued a specialized document: Action Plan for Strengthening Reserve Talent Cultivation in Gymnastics (2025–2028). In July 2025, the Gymnastics Management Center of the General Administration of Sport and the Jiangxi Provincial Sports Bureau jointly signed the Strategic Cooperation Agreement on Promoting the High-Quality Development of the Mass Gymnastics Program in Jiangxi Province, translating core academic theories into concrete action plans. Meanwhile, in response to the pain point that gymnastics has become “divorced from daily life,” relevant literature has put forward the idea of “gymnastics for daily living.” It stresses that gymnastics is not only a competitive sport but also a cultural activity that enhances life skills and shapes healthy lifestyles. This theoretical shift has provided direct guidance for the initiative of “bringing gymnastics into government agencies, communities, and families” [15]. Moreover, high quality journals have applied artificial neural networks and intelligent optimization algorithms to decompose, calculate, and adjust parameters in gymnastics movements, delivering evidence-based training guidance for gymnasts [16^],^ Furthermore, via vision-semantic aligned temporal analysis networks, feature alignment and structural parsing of movement sequences are conducted to realize objective quantitative assessment of technical maneuvers [17], fundamentally driving the transformation of training practice from experience-guided to data-driven.

#### 1.1.3. Gymnastics research literature serves as a valuable carrier for the inheritance and innovation of gymnastics culture.

Gymnastics research published in high quality journals not only dissects gymnastic techniques and movements but also embodies the sporting spirit of pursuing excellence and striving for perfection.Some studies have introduced the theoretical tool of the “cultural onion model” to deconstruct gymnastics culture into layers including symbols, heroic figures, rituals, and values, providing a scientific theoretical framework for the systematic inheritance of gymnastics culture [18]. Some studies have summarized the spiritual traits of Chinese gymnastics culture as “a belief in diligence rewarded, a dedication to excellence, a commitment to daring innovation, and an inclusive openness to diverse influences.” These qualities have endowed gymnastics culture with a distinct national spirit [19]. By further exploring the profound spiritual connotations of Chinese gymnastics, this research responds academically to the Guiding Opinions on Further Strengthening the Standardized Development of Sports Culture [20], which calls for in-depth excavation of the spiritual essence inherent in individual sports.Meanwhile, against the backdrop of national strategies such as the Healthy China initiative and Sports Power strategy, gymnastics has expanded its cultural values from “striving for competitive glory” to “serving national health and promoting all-round human development.” Drawing on systematic research from scientific, sociological, and educational perspectives, the literature demonstrates the cultural and social functions of gymnastics beyond competitive sport itself, laying a solid theoretical foundation for this transformative shift. By developing diversified curricula and evaluation systems, studies offer practical operational guidelines for the transition of gymnastics from elite competition to public health promotion. Through the production and dissemination of academic discourse, they reshape stereotypical perceptions of gymnastics among society, the media, and policymakers. This in turn elevates gymnastics to a popular sports and cultural carrier with profound cultural heritage and social functions, highlighting its unique role in strengthening the national physique and inspiring the national spirit.

## 2. Theoretical framework

In composite competitive sports such as gymnastics, certain practically-valuable research Topics fall into a structural state of low coverage and low concentration due to high research barriers. Assessing research value solely by publication output may easily overlook the academic significance of these Topics. To address this, the present study develops a two-dimensional diagnostic framework of “Topic concentration-Topic coverage” (see Fig 1). Drawing on the internal structure of knowledge production, it derives differentiated development paths and establishes a complete logical chain from empirical findings to research recommendations.

**Fig 1:**
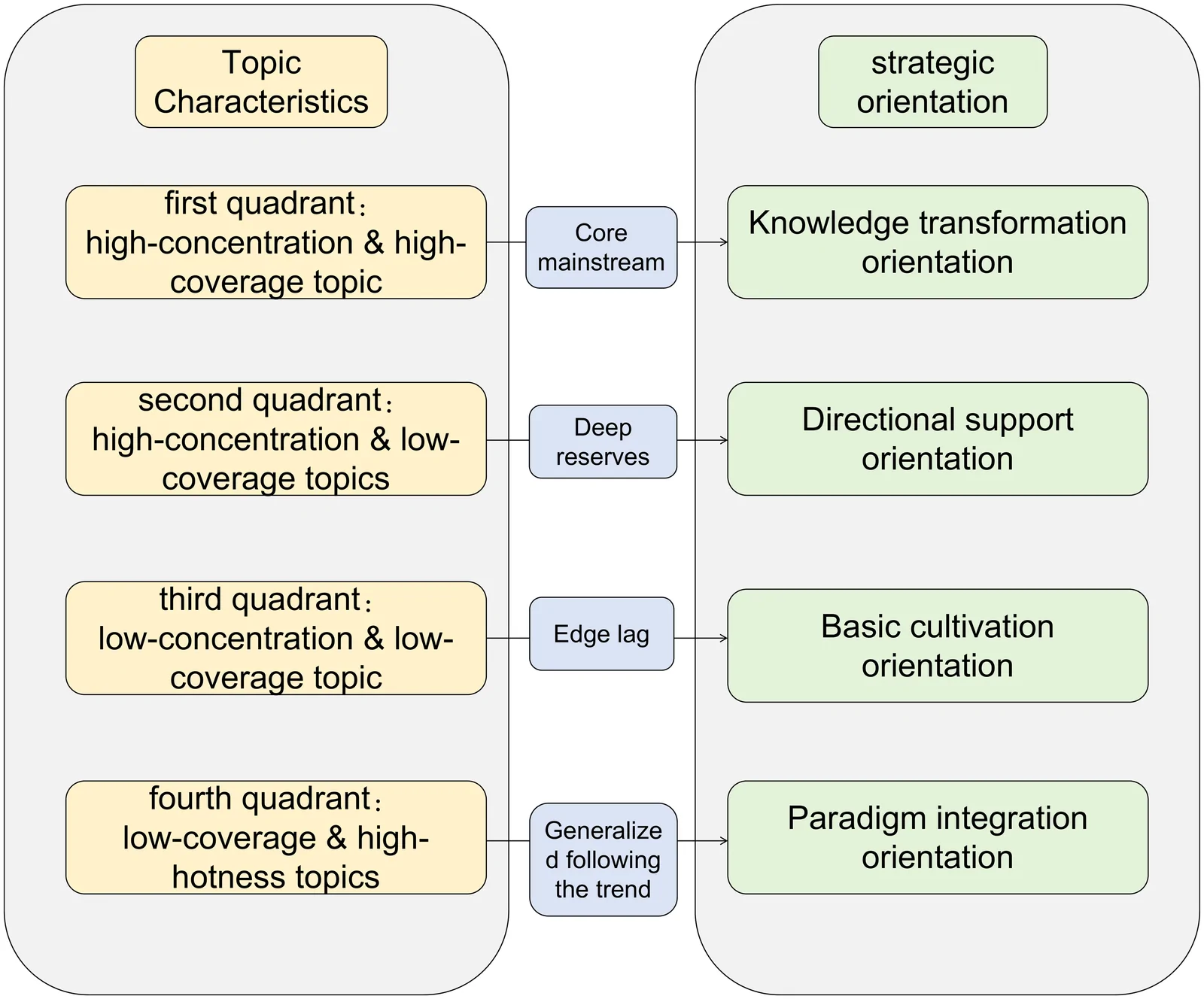
Two-dimensional knowledge-structure diagnostic matrix based on Topic concentration and Topic coverage.

## 3. Topic mining of Chinese gymnastics research literature based on latent dirichlet allocation model

### 3.1. Research methods

The Latent Dirichlet Allocation (LDA) model is an unsupervised probabilistic topic model developed by introducing a Dirichlet Distribution prior on the basis of Probabilistic Latent Semantic Analysis (PLSA). It characterizes the semantic relationships between topics and words within documents through a three-layer Bayesian structure of document-topic-word,Proposed by David Blei et al. in 2003 [21], this model can effectively extract latent topics from large-scale document collections and corpora. With strong dimensionality reduction, modeling, and scalability capabilities, it serves as a core tool in natural language processing (NLP) and text mining.Since the LDA model performs computations via probabilistic inference algorithms, and its computational efficiency is independent of the number of documents, it can handle large-scale text data [22],It has been applied in policy text metrology [23],library and information science [24],web text analysis [25] and other related fields.

The number of topics (K) is the core hyperparameter of the LDA model,This study integrates multi‑criteria decision‑making and objective weighting methods to systematically determine the optimal number of topics, and conducts qualitative validation through manual review of the semantic coherence of high-frequency feature words within each topic as well as the discrimination across different topics. First, statistical fitting indicators are calculated for topic numbers ranging from 2 to 30, including the Akaike Information Criterion (AIC), Bayesian Information Criterion (BIC), and log-likelihood value, to evaluate the model’s explanatory power over the data; Then, the Jensen-Shannon Divergence (JSD) is introduced as a quantitative measure of topic distinctiveness. For convenient subsequent comprehensive comparison, JSD is normalized to the interval [0, 1], and its complementary value 1-JSD_normalized is directly defined as the topic overlap score, ensuring intuitive interpretability of the indicator.To automatically and objectively identify the core indicators with genuine discriminatory power in the multi‑indicator system and assign weights precisely, this study introduces the entropy weight method as an objective weighting technique. Weights are automatically calculated based on the inherent variation (information entropy) of each indicator across different topic numbers, with higher weights assigned to indicators showing greater variation. First, forward and negative indicators are subjected to unification and standardization processing, followed by calculating the entropy value and difference coefficient for each indicator. Subsequently, the objective weight of each indicator is derived, and the comprehensive score for each candidate topic number is computed. The topic number corresponding to the highest comprehensive score is selected as the optimal solution. When calculating JSD, Inf or NA values may occur for a small number of topic candidates due to the boundary conditions of the probability matrix. In this study, the neighborhood-mean interpolation method is adopted for correction, whereby outliers are replaced with the arithmetic mean of valid JSD values of adjacent topic numbers. To verify that this treatment does not compromise the robustness of conclusions, sensitivity analysis is supplemented. Missing JSD values are processed using neighborhood-mean interpolation and complete-case deletion (removing topic numbers with outliers) respectively, and the recommended optimal topic number is recalculated for both approaches. The consistent results obtained from the two methods demonstrate that outlier handling exerts no substantial effect on model conclusions. Reasonable candidates for topic numbers are thus preliminarily identified. Afterwards, members of the research team manually examine the top-10 high-frequency feature words of each topic, together with the titles and abstracts of three representative documents with the highest probabilities, to evaluate the semantic coherence and interpretability of each topic, as well as the content discrimination among different topics.

### 3.2. Data sources and preprocessing

Through advanced search on the China National Knowledge Infrastructure (CNKI, domestic version), the time window for literature retrieval was set from 2016 to 2025, the Chinese search term “Ticao” (corresponding to the English term “gymnastics”) was adopted, and the search field was set to “topic,” which covers titles, keywords and abstracts simultaneously. Within CNKI’s Chinese-language retrieval environment, “Ticao” serves as a hypernym and automatically matches all compound terms containing this Chinese character string, such as “competitive gymnastics” and “rhythmic gymnastics.” Meanwhile, given the indexing conventions for Chinese sports-discipline literature, papers on related fields including aerobics and aerobic gymnastics frequently mention the concept of “Ticao” in their titles, abstracts or keywords, and will therefore also be captured by this search term. Data collection was concluded on December 30, 2025, and literature types were restricted to SCI-source journals, PKU Core (Peking University Core), and CSSCI. A total of 458 relevant records were retrieved. To maintain the homogeneity of the technical system, focus on the research scope, and follow the conventions of existing academic studies, only literature related to rhythmic gymnastics, aerobics, and artistic gymnastics was selected, while studies on cheerleading, setting-up exercises, and other related fields were excluded. Documents with “gymnastics” in their titles were extracted, and the remaining literature was manually screened by consensus for gymnastics-specific content using keywords such as “Uneven Bars,” “Vault,” “Horizontal Bar,” and “Balance Beam.” Ultimately,169 high quality journal papers were obtained, including 30 in 2016, 26 in 2017, 14 in 2018, 21 in 2019, 26 in 2020, 11 in 2021, 11 in 2022, 6 in 2023, 15 in 2024, and 9 in 2025. Given the clear and operable screening criteria-publications dated 2016–2025, published in high-quality Chinese journals indexed by CNKI, and explicitly Topicd on gymnastics-subjective judgment was kept to a minimum. Accordingly, literature screening was independently completed by the first author. To guarantee screening quality, the co-first author reviewed the final set of 169 included papers to confirm that each met all three criteria above. In addition, the corresponding author randomly sampled 20% of the excluded literature for re-examination to verify the reasonableness of exclusion decisions.

English abstracts were saved as research objects. and the data-processing workflow is illustrated in Fig 2. Meanwhile, to ensure the replicability of the study, digital object identifiers (DOIs) of the literature were retained. Verification of textual data can be performed by accessing “Advanced Search” on CNKI (domestic version), selecting “Professional Search,” setting “AB = Abstract” or “DOI = DOI” as retrieval fields, and inputting the saved text data.

**Fig 2:**
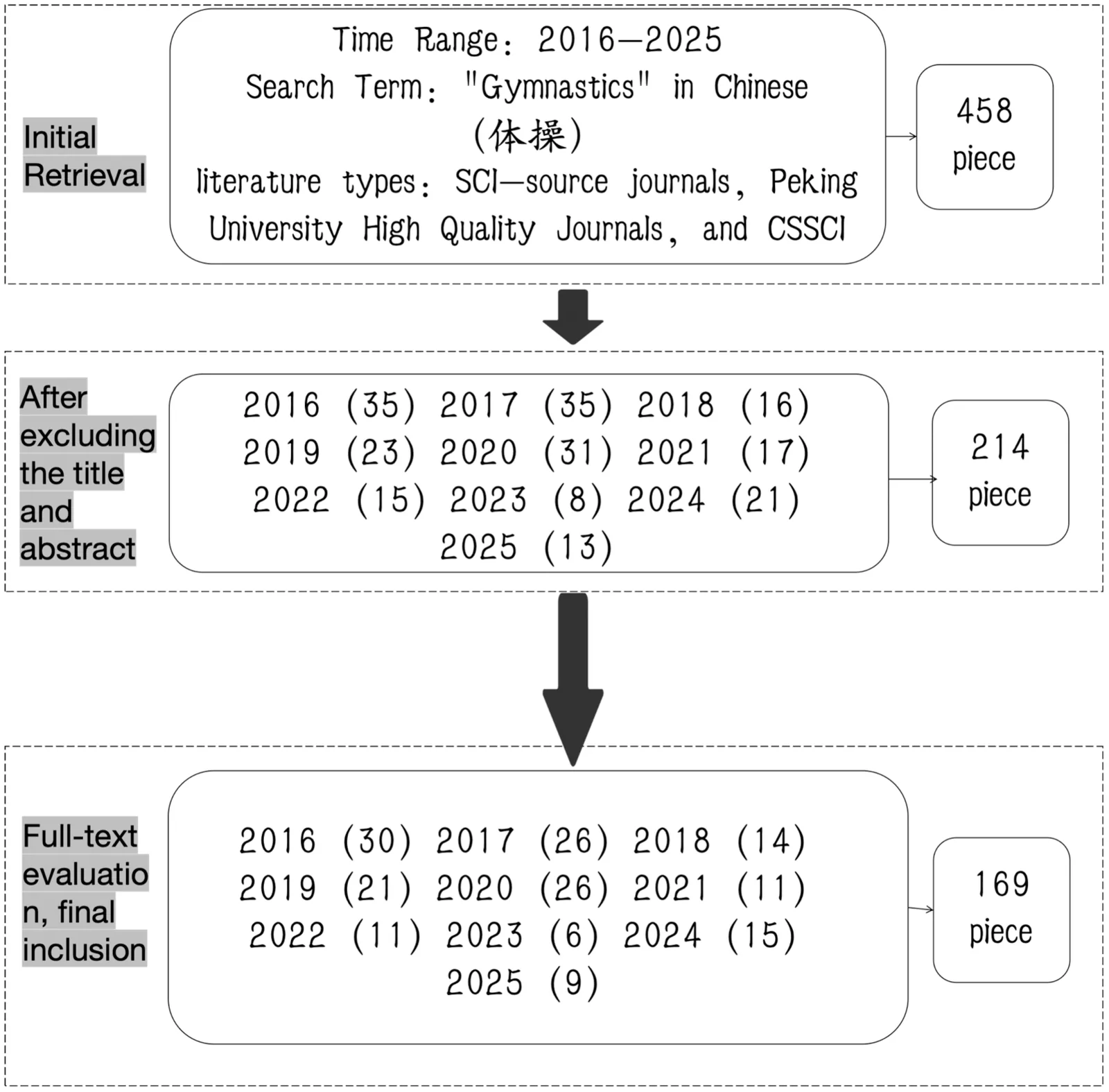
Data-processing workflow.

To improve the accuracy and reliability of analytical results, a systematic text preprocessing procedure was employed to refine textual data quality. First, case variations were eliminated through corpus standardization, and a multi-level noise removal scheme was applied to sequentially eliminate punctuation marks, numerals, and customized stop words. Specifically, the standard stop-word list from the tm package was used first, and an additional 171 domain-specific stop words were supplemented, including research methods, numerals, and adverbs, to remove meaningless interference for topic analysis. Second, lemmatization was adopted for lexical standardization and redundant space removal, and a closed-loop cleaning mechanism was established for secondary filtering. Finally, the constructed document-term matrix provided a high-quality data structure for subsequent topic modeling, effectively guaranteeing the reliability of analytical outcomes.

### 3.3. Topic mining of gymnastics-related research literature in chinese high quality journals

#### 3.3.1. Optimal topic number selection based on multi-criteria decision-making and objective weighting methods.

All analyses were performed in R 4.4.1. LDA modeling was mainly implemented via the ‘topicmodels’ package (version 0.2.17), with parameter estimation conducted using the default Variational Expectation-Maximization (VEM) algorithm. A random seed of 1234 was set during model fitting to guarantee result reproducibility. Both hyperparameters α and β adopted the default settings of the `topicmodels` package (α was estimated adaptively under the VEM algorithm, and β took the default value of 0.1). The selection of the optimal number of topics essentially seeks an optimal trade-off between model parsimony and complexity. Model-comparison metrics including AIC, BIC and Log-Likelihood, together with the content-comparison metric Jensen-Shannon Divergence, were combined. The entropy weight method was further introduced to construct a comprehensive evaluation value for determining the optimal topic number. Meanwhile, considering multiple factors, the candidate range of topic number K in this study was set to all positive integers from 2 to 30. Unreasonable settings such as K = 1 (lack of thematic structure) and K > 30 (excessive parameterization) were excluded to guarantee the model’s practical interpretability.

As shown in Fig 3, the topic number K corresponding to the minimum AIC is 7, and that corresponding to the minimum BIC is 3. By calculating the log-likelihood difference between adjacent topic numbers, the growth saturation point is determined when the absolute difference value falls below the relative threshold for the first time, corresponding to a topic number K of 17. Since the optimal topic numbers derived from each metric differ, the Jensen-Shannon Divergence measure was further calculated.

**Fig 3:**
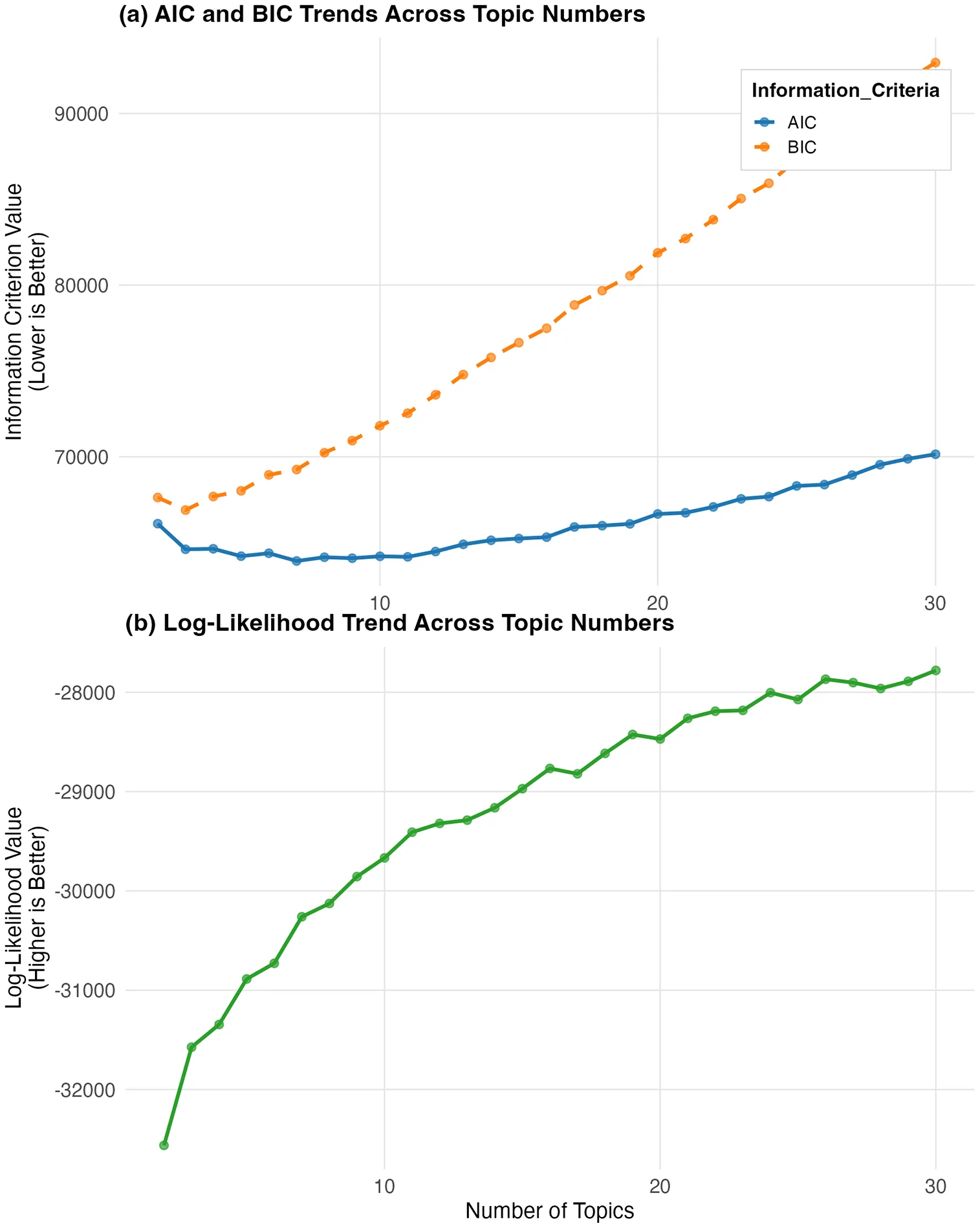
Line plots of AIC, BIC, and log-likelihood for 2-30 topics.

Fig 4 (top-left) illustrates the trend of JSD with varying topic numbers, where a larger value indicates better topic discrimination. Fig 4 (top-right) presents the trend of topic overlap scores, where a smaller value represents lower topic overlap. Fig 4 (bottom-left) presents the comparison of candidate topic numbers, and Fig 4 (bottom-right) provides a comprehensive comparison between JSD and overlap scores. It is finally obtained that when the topic number K is 30, the JSD value reaches the highest at 0.591 and the overlap score is the lowest at 0.000. Accordingly, the topic discrimination is maximized at K = 30. Since the optimal topic numbers derived from AIC, BIC, log-likelihood and JSD are inconsistent, the entropy weight method is adopted to conduct objective weighting and comprehensive integration of the four indicators-AIC, BIC, log-likelihood and JSD-so as to scientifically determine the optimal topic number for the LDA model. The calculation results show that BIC is assigned the highest weight (0.373), providing the greatest amount of information in distinguishing models with different topic numbers; followed by AIC (0.294); while the weights of log-likelihood (0.181) and JSD (0.151) are relatively lower. The weighted comprehensive scores of topic numbers K (2–30) are compared (retaining three decimal places), and the top 10 topics with the highest comprehensive scores from the entropy weight method are listed in Table 1:

**Table 1:** Top 10 topics ranked by comprehensive score of the entropy weight method.

Topic	Entropy Weight Method Comprehensive Score	Topic	Entropy Weight Method Comprehensive Score
Topic 7	0.802	Topic 9	0.801
Topic 11	0.797	Topic 10	0.794
Topic 8	0.789	Topic 12	0.767
Topic 5	0.765	Topic 6	0.763
Topic 13	0.738	Topic 14	0.729

**Fig 4:**
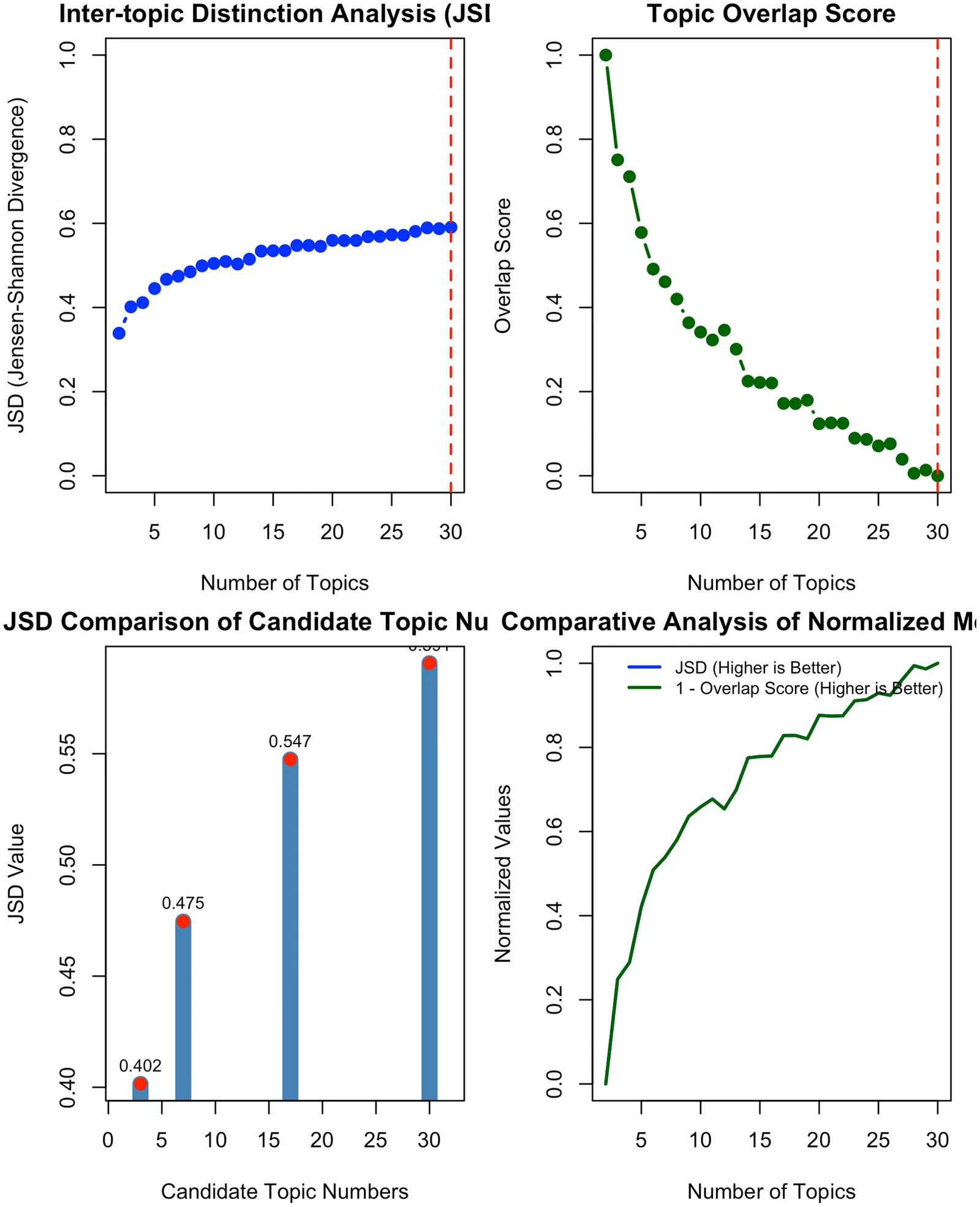
Jensen-Shannon Divergence (JSD) trend chart.

As shown in Table 1, when the topic number K is 7, the comprehensive score of the entropy weight method achieves the highest evaluation value (0.802) among all candidate models (K = 2–30). This result indicates that the 7-topic scheme strikes the optimal balance between model simplicity, goodness of fit, and topic distinguishability. As the entropy weight method objectively integrates the information content and variation degrees of the four indicators-AIC, BIC, log-likelihood, and JSD-it represents a robust comprehensive evaluation approach. Accordingly, this study finally determines that the optimal topic number K for Chinese gymnastics-related high quality journal papers from 2016 to 2025 is 7.

#### 3.3.2. Topic extraction of gymnastics research literature in Chinese high quality journals.

The Latent Dirichlet Allocation (LDA) model was applied to textual data using the optimal number of topics, yielding the topic-word distribution results. The top 10 topic words in the retained Topics 1–7 were organized, and prominent terms among high-probability feature words were identified. Team members examined the top-10 high-frequency feature words of each topic, as well as the titles and abstracts of three representative documents with the highest probabilities. The results demonstrated that all seven topics exhibited coherent and interpretable semantics with clear content boundaries between one another, which validated the rationality of the seven-topic solution. Subsequently, two researchers (the first author and the co-first author) independently reviewed the high-frequency words and assigned labels to each topic with reference to the original abstract samples. Discrepancies were resolved through discussion, and the final labels were reviewed and confirmed by the corresponding author.

As shown in Table 2, Topic 1 refers to the technical analysis of bar connections and choreography in gymnastics competitions, focusing on technical examination and scoring research of bar movement combinations and arrangements in competitive events. Topic 2 covers the evolution of the team pattern in men’s world artistic gymnastics, concentrating on team competitive strength, global competitive landscape and national performance analysis of male artistic gymnasts in major tournaments. Topic 3 involves the biomechanical analysis of landing movements among gymnasts, exploring differences in peak performance exhibited by individuals of various ages when executing landing motions and their correlation with injury risks. Topic 4 addresses research on curriculum and instructional practice in school gymnastics education, with emphasis on content design, system construction and teaching effectiveness of school gymnastics courses. Topic 5 presents a study on balance capability in gymnasts’ apparatus movements, centering on balance control and movement characteristics displayed by gymnasts while performing apparatus routines. Topic 6 provides technical movement analysis of elite athletes, conducting detailed examinations of elite gymnasts’ techniques from the perspectives of force characteristics, angular parameters and body posture. Topic 7 focuses on the development of reserve talents in competitive gymnastics, including the evolution of talent development systems, resource integration and managerial experience.

**Table 2:** The distribution of topic-high probability feature word.

Topic	Topic Label	High-Probability Feature Words	Representative Documents (Top 3 by Probability)	Topic Probability Distribution
Topic 1	Technical Analysis of Bar Connections and Choreography in Gymnastics Competitions	score, bar, connection, world, arrangement, championship, technical, olympic, athlete, exercise	2023−010.txt 2017−066.txt 2019−028.txt	0.996 0.994 0.993
Topic 2	Evolution of the Team Pattern in Men’s World Artistic Gymnastics	team, olympic, artistic, competition, man, strength, world, tokyo, international, country	2022−010.txt 2021−026.txt 2018−031.txt	0.996 0.993 0.991
Topic 3	Biomechanical Analysis of Landing Movements in Gymnasts	land, exercise, peak, difference, injury, increase, test, age, experimental, perform	2016−036.txt 2019−035.txt 2022−024.txt	0.991 0.991 0.990
Topic 4	Research on Curriculum and Teaching Practice in School Gymnastics Education	education, physical, school, teach, content, system, dance, curriculum, student, research	2020−04.txt 2022−09.txt 2023−09.txt	0.992 0.992 0.989
Topic 5	Study on Balance Ability of Gymnasts in Apparatus Movements	athlete, action, ability, balance, feature, complete, individual, datum, beam, apparatus	2017−075.txt 2024−022.txt 2021−028.txt	0.990 0.985 0.952
Topic 6	Movement Technique Analysis of Elite Athletes	strength, angle, elite, muscle, support, rotation, health, athlete, relationship, position	2020−047.txt 2019−048.txt 2020−029.txt	0.993 0.993 0.991
Topic 7	Cultivation of Reserve Talents in Competitive Gymnastics	talent, cultural, resource, reserve, evolution, system, element, national, management, experience	2024−09.txt 2016−014.txt 2017−072.txt	0.990 0.989 0.984

## 4. Strategic value matrix and quadrant distribution of gymnastics research topics in Chinese high quality journals

### 4.1. Construction of value evaluation dimensions for gymnastics research topics in Chinese high quality journals

#### 4.1.1. Topic concentration.

The concentration of a topic is reflected by the topic strength indicator. First, the topic-word log-probability matrix was extracted from the converged LDA model. Second, exponential operations were performed on the matrix values to convert them into standard conditional probabilities. Third, these probabilities were sorted in descending order, and the top 10 terms were selected. Finally, the probability values of the 10 core terms belonging to the same topic were summed, and the total obtained was defined as the topic strength value. A strength threshold was set based on the average of all topic strength values, and topics above this threshold were classified as high-intensity topics. A strength value closer to 1 indicates that the probability mass of the topic is concentrated in a small number of core terms, with clear topic boundaries, strong cohesion, and high interpretability. A lower strength value suggests scattered topic concepts and vague semantics.

#### 4.1.2. Topic dispersion.

The dispersion of a topic is reflected by the topic popularity indicator. First, the number of documents assigned to each topic by the LDA model in the dataset was counted. Second, the document frequency index, expressed as the percentage of literature affiliated with each topic relative to the total number of documents in the corpus, was used as a quantitative basis for evaluating the academic visibility and thematic salience of the topic in existing research. Third, the average number of documents across all topics was calculated as a benchmark threshold to distinguish high-popularity topics from low-popularity topics. By comparing the document count of each topic with the average value, topics with a document number higher than the average were identified as hot topics, representing research directions that have received greater attention in current studies.

### 4.2. Construction of a four-quadrant matrix for gymnastics research topics in Chinese high quality journals

In this study, the topic popularity value was set as the X-axis, and the topic intensity value as the Y-axis, with the mean intensity and mean popularity serving as quadrant dividing lines. Meanwhile, a four-quadrant framework was established with the two means as the coordinate origin.Topics in the first quadrant satisfy both intensity≥mean and popularity≥mean. Topics in the second quadrant satisfy intensity≥mean but popularity < mean. Topics in the third quadrant satisfy both intensity < mean and popularity < mean. Topics in the fourth quadrant satisfy intensity < mean but popularity≥mean. Finally, each topic is assigned to the corresponding quadrant according to its coordinate values, this study intuitively presents the structural position of each research topic in knowledge production, providing descriptive references for future research directions.

As shown in the Topic Intensity-Popularity Four-Quadrant Analysis Chart (Fig 5), the popularity threshold is 24.1 and the intensity threshold is 0.38. Among the extracted topics: Topic 4 (Research on Curriculum and Teaching Practice in School Gymnastics Education) falls into the first quadrant as a high-concentration & high-coverage topic. Topic 5 (Study on Balance Ability of Gymnasts in Apparatus Movements) and Topic 6 (Movement Technique Analysis of Elite Athletes) belong to the second quadrant as high-concentration & low-coverage topics. Topic 1 (Technical Analysis of Bar Connections and Choreography in Gymnastics Competitions) is located in the third quadrant as a low-concentration & low-coverage topic. Topic 2 (Evolution of the Team Pattern in Men’s World Artistic Gymnastics), Topic 3 (Biomechanical Analysis of Landing Movements in Gymnasts) and Topic 7 (Cultivation of Reserve Talents in Competitive Gymnastics) fall into the fourth quadrant as low-coverage, high-hotness topics.

**Fig 5:**
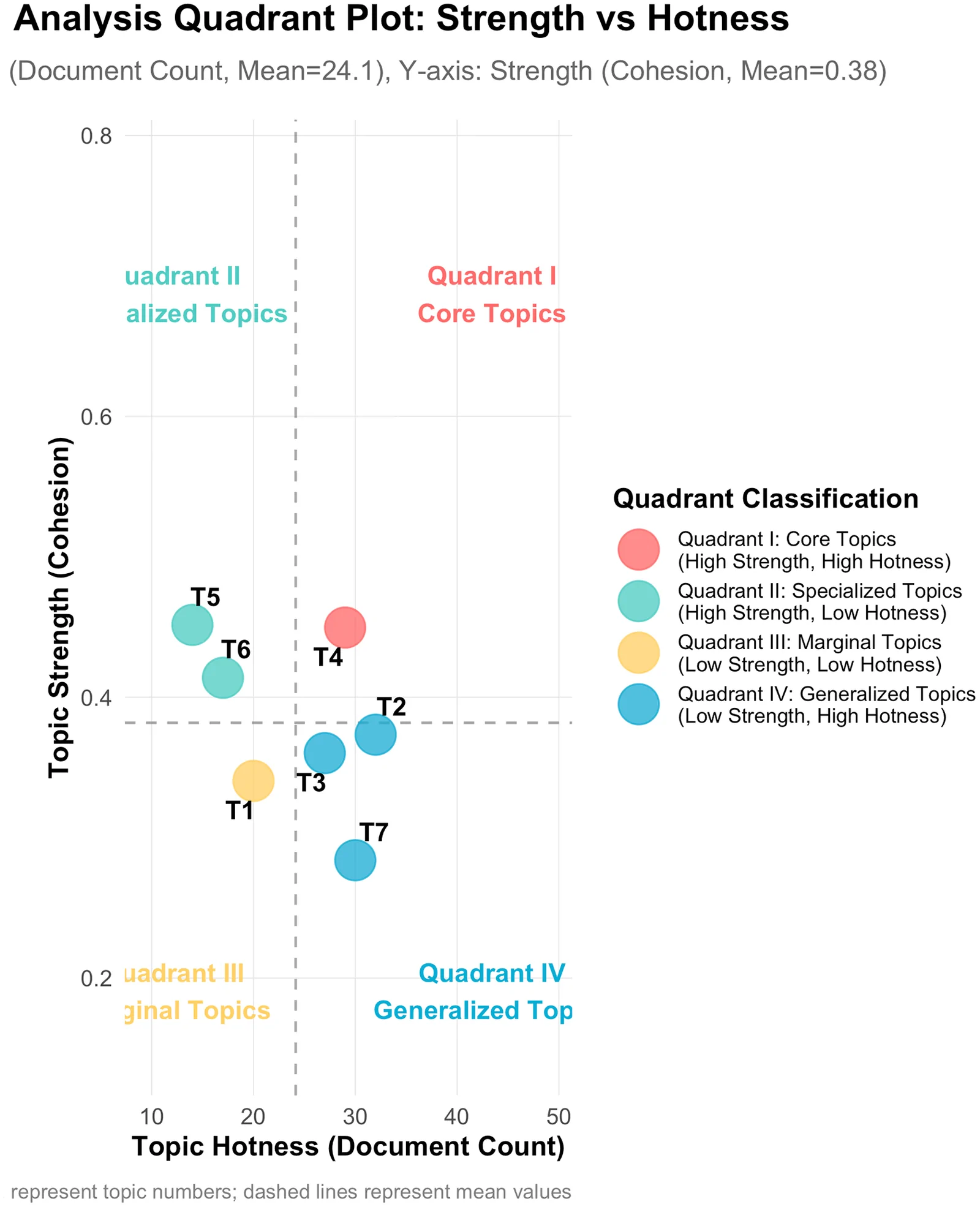
Topic intensity-popularity four-quadrant analysis chart.

It can be concluded from the above research that Topic 4 (research on curriculum and teaching practice in school gymnastics education) serves as the most widely concerned and fully accumulated core issue in the knowledge production of gymnastics research in high-quality journals. Centering on gymnastics curriculum design and teaching implementation in school scenarios, this topic focuses on key research directions such as gymnastics education and curriculum design. Its abundant knowledge accumulation lays a solid literature foundation for the subsequent transformation of academic achievements into practical teaching guidelines, teacher training frameworks and curriculum standard references.Topic 5 (research on balance ability of gymnasts in apparatus movements) and Topic 6 (movement technical analysis of elite gymnasts) belong to the topic type with focused research focuses but limited research coverage. The keywords of the two topics are highly concentrated on the biomechanical analysis of specific technical movements and balance control mechanisms, indicating that relatively stable academic consensus has been formed in these niche research fields. Nevertheless, the overall publication volume is low and the participating researchers are relatively centralized. Accordingly, the balance ability of apparatus movements and the technical analysis of elite athletes are research fields with high research depth but insufficient diffusion in current gymnastics knowledge production. Topic 1 (technical analysis of bar connection and choreography in gymnastics competitions) is categorized as a type of dispersed research with limited hotness. The keywords of this topic are distributed discretely without clear core conceptual focus. Meanwhile, its overall literature scale is small, indicating that no stable research paradigm has been formed for this research issue in academic circles, resulting in insufficient knowledge supply. Therefore, the technical analysis of bar connection and choreography is an underexplored niche direction in current gymnastics knowledge production. Topic 2 (evolution of the team pattern in world men’s artistic gymnastics), Topic 3 (biomechanical analysis of gymnasts’ landing movements), and Topic 7 (talent cultivation for competitive gymnastics reserves) are classified as topics with high hotness yet dispersed research. These topics possess a large volume of literature, yet their keywords show discrete distribution and lack definite core conceptual focus, which indicates that relevant research has not formed a unified analytical framework or theoretical paradigm. It is thus concluded that these research issues are still in the stage of multi-perspective exploration.

## 5. Differentiated development paths of gymnastics research topics in Chinese high-quality journals based on topic quadrant classification

### 5.1. Focus on advantageous fields and deepen the theoretical and practical transformation of topics with high coverage and high concentration

The national top-level design document, Opinions on Deepening Integration of Sports and Education to Promote the Healthy Development of Young People [26] clearly puts forward the requirement to “establish the educational concept of health first” and emphasizes “carrying out rich and colorful after-school training and competition activities”; The educational programmatic document, Opinions on Comprehensively Strengthening and Improving School Physical Education in the New Era [27] emphasizes the need to continuously deepen teaching reform and actively improve the evaluation mechanism; Meanwhile, the Compulsory Education Physical Education and Health Curriculum Standards (2022 Edition) [28] identifies “gymnastic sports” as an important component of specialized motor skills; National strategies, educational guidelines and curriculum standards together constitute the policy basis and institutional guarantee for promoting the reform of school gymnastics teaching. Moreover, Topic 4 (research on curriculum and teaching practice in school gymnastics education) lies at the intersection of multiple policy objectives, including the national health strategy, educational evaluation reform, and the in-depth integration of sports and education, presenting practical urgency for teaching reform. The current academic outputs of such topics with high coverage and high concentration present a high degree of homogeneity. Therefore, it is necessary to systematically integrate and meta-analyze existing research achievements, and extract consensus conclusions on the curriculum and teaching practice of school gymnastics education. This approach helps clarify the knowledge boundaries and identify methodological limitations of cumulative research, thereby providing a clear starting point for subsequent studies and avoiding low-level repetitive research. Furthermore, this study identifies the methodological deficiencies of existing literature in terms of research design, sample selection, and measurement tools, clarifies the key research directions and scientific issues that need in-depth exploration in this field, and promotes the reflection and innovation of the research paradigm. Finally, it optimizes the dissemination form of academic achievements and facilitates in-depth academic communication within the research community.

### 5.2. Explore and deepen in-depth research fields to expand the practical application scenarios of topics with low coverage and high concentration

Topics (research on balance ability of gymnasts in apparatus movements) and (movement technical analysis of elite gymnasts) fall into the quadrant of low-coverage and high-concentration. Hu Haixu et al. pointed out that, as a cutting-edge innovative direction in sports science, domestic research on sports performance analysis is relatively limited and mainly focuses on individual sports events [29]. Their study suggested that multi-dimensional insights from performance analysis should be transformed into targeted training adaptations to construct a structured performance analysis ecosystem. This conclusion provides a direct methodological reference for expanding the application scenarios of specialized technical gymnastics topics in this quadrant. Firstly, it is essential to refine the consensus of specialized technical research and establish a transformative research agenda. Drawing on the research approach adopted by Torrès-Salinas et al.[30], who revealed the distribution of collaboration potential in sports science via advanced topic modeling, existing highly-focused findings under topics in this quadrant (e.g., balance control of apparatus movements and technical analysis of elite gymnasts) can be systematically integrated. Core scientific issues and methodological consensus can be identified to formulate a future research agenda oriented toward training-practice references. Secondly, emerging technical methods may be introduced to expand the connection scenarios for training-oriented research. By adopting emerging technologies such as wearable inertial sensing [31], high-speed motion capture [32] and artificial-intelligence-based video analysis [33] specialized technical analysis can be transformed from qualitative description toward data-driven quantitative diagnosis, so as to expand its application scenarios in training practice. Furthermore, the in-depth integration of performance analysis and event-specific training should be promoted. Pan indicated that competitive sports science is undergoing a paradigm shift from reductionism to complex systems, and future frontiers will focus on the in-depth integration of biomedical technologies and humanistic psychology [34]. Inspired by this viewpoint, topics in this quadrant should break through the limitations of a single biomechanical perspective. Multiple dimensions including neuromuscular control, motor cognition and training load management can be incorporated into the analytical framework for elite gymnastics techniques, so as to construct a systematic technical performance analysis model.

### 5.3. Focus on potential fields and explore the basic research value of low-coverage and low-concentration topics

Topic 1 (technical analysis of bar connection and choreography in gymnastics competitions) is a topic with low concentration and low coverage. It is characterized by limited literature scale and dispersed research foci, and no stable research paradigm has been established. Nevertheless, it bears the under-exploited basic-research value within the knowledge landscape of the gymnastics discipline. Wang Jiahong et al.[35] noted that the real-world dilemma of “low hotness for niche disciplines"still exists within China’s current sports discipline system. Nevertheless, the construction of the sports discipline system requires attention to the main research directions of branch disciplines such as basic and niche subjects. This judgment provides disciplinary-system-level evidence for exploring the basic-research value of gymnastics research topics in this quadrant. First, identify core scientific issues through systematic literature tracing. This study systematically reviews and critically integrates existing literature on the technical analysis of bar connection and choreography in gymnastics competitions. It identifies the core conceptual frameworks, key variable relationships, and methodological limitations of relevant studies, and refines the basic positioning and core scientific problems of this topic in the knowledge system of gymnastics discipline, so as to provide conceptual anchors for subsequent research. Second, reinforce the design and methodological foundation of weak research. By identifying the deficiencies of existing studies in sample selection, measurement tools, research design, and theoretical perspectives, future research can further clarify standardized pathways in experimental design, data collection, and analytical techniques. The introduction of diverse theoretical perspectives and research methods such as sports biomechanics and exercise physiology can improve the methodological rigor of relevant research. Third, promote interdisciplinary theoretical dialogue and explore disciplinary growth points. Breaking through the limitations of the single technical analysis perspective, topics in this quadrant can integrate diversified theoretical perspectives and explore potential theoretical contributions to the future research frontiers of sports science.

### 5.4. Integrate scattered topics to promote the knowledge systematization of high-coverage and low-concentration topics

Topics (evolution of the team pattern in world men’s artistic gymnastics), (biomechanical analysis of gymnasts’ landing movements), and (talent cultivation for competitive gymnastics reserves) fall into the high-coverage and low-concentration quadrant. Although a certain body of research outputs has been accumulated for such topics, knowledge is widely and dispersedly distributed without a consolidated core paradigm. Systematic integration is urgently required to advance their knowledge systematization. Tan Guangxin et al.[36] pointed out that to construct the independent knowledge system of Chinese sport science, researchers should follow the internal logic of “independence-knowledge-system,” and systematically explore the generation mechanism and construction path of the knowledge system from dimensions including knowledge origins, research paradigms, iconic concepts, and research methods. This provides disciplinary-level theoretical evidence for the knowledge systematization of topics in this quadrant. To promote the knowledge systematization of topics in this quadrant, first, it is necessary to integrate scattered fragmented findings and construct a comprehensive conceptual framework. Through systematic review or meta-analysis, empirical conclusions dispersed in extensive literature can be integrated to refine a comprehensive conceptual model for these generalized topics, providing a unified cognitive foundation for subsequent research.Second, clarify the boundaries of core scientific issues within generalized research topics. From the macroscopic perspective guided by scientific knowledge mapping, this study identifies key scientific issues that are frequently mentioned but insufficiently discussed, defines corresponding research boundaries, and avoids the dispersion of research resources. Finally, promote interdisciplinary theoretical dialogue to facilitate knowledge systematization. Researchers with diverse disciplinary backgrounds are encouraged to conduct academic dialogues on common research topics, integrate scattered theoretical perspectives into a more explanatory knowledge system, and further improve the academic depth of research topics in this quadrant.

## 6. Conclusion

Based on the two-dimensional matrix of concentration-coverage, this study constructs a four-quadrant diagnostic framework to classify gymnastics research literature from Chinese high-quality journals and diagnose their structural patterns. It reveals the position of each Topic in disciplinary knowledge production and puts forward differentiated development paths accordingly. The research show that current gymnastics research in Chinese high quality journals presents clear structural differentiation and strategic layout. high-concentration and high-coverage topic focus on the strategy of sport-education integration, with the core aim of solving practical problems in school gymnastics education; high-concentration and low-coverage topics focus on the Olympic glory strategy, dedicated to cutting-edge research such as elite technical biomechanics; low-concentration and low-coverage topic concentrated on rule adaptation and tactical adjustment, involving rapid response and optimization under regulatory constraints; low-coverage & high-hotness topics are aligned with the social extension of the sports power strategy, addressing macro issues including reserve talent development and the international competitive landscape. Findings indicate that gymnastics research in Chinese high quality journals has formed a comprehensive knowledge production ecosystem that both serves the cutting-edge competitiveness of China’s national gymnastics team and is rooted in the social foundation and educational system. Based on the four-quadrant diagnostic results derived from the two-dimensional matrix of concentration-coverage, this study proposes differentiated development paths for various types of topics: topics with high coverage and high concentration are oriented toward “deepening theoretical and practical transformation”; topics with low coverage and high concentration aim to “expand practical application scenarios”; topics with low coverage and low concentration focus on “exploring the value of basic research”; and topics with high coverage and low concentration target “promoting knowledge systematization.” This classification framework elevates computational text analysis from a mere technical tool to an analytical framework for revealing knowledge structures and the status of disciplinary development, advancing the interdisciplinary integration of bibliometrics and sports discipline system research. The findings provide a literature-evidence-based analytical procedure and descriptive reference for mapping the knowledge landscape of gymnastics discipline and selecting future research directions. They also offer methodological implications for topic evaluation in other sub-fields of sports science.

Naturally, this research has certain limitations. Although high quality journal literature reflects cutting-edge exploration and consensus within the academic community and serves as a key source of high-quality, structured knowledge, the study does not cover dissertations, conference proceedings, and other document types. Future research will incorporate multi-source heterogeneous data to construct a more comprehensive knowledge graph.

## Supporting information

S1 File Custom stopwords used in the text mining analysis. This file lists all custom stopwords defined and applied for text preprocessing in this study.
(PDF)
