# Supplementary material for: Quadrant classification and development path of gymnastics-related research topics in Chinese high-quality journals: Based on SCI, PKU Core and CSSCI source journals
Source: PLoS One. 2026 Nov 2;21(11):e0355670. doi: 10.1371/journal.pone.0355670 (PMC13726921; doi:10.1371/journal.pone.0355670)
Supplement: Supplementary file 1 [file pone.0355670.s001.pdf]

```
# 定义自定义停用词列表
custom_stopwords<-c(stopwords("en"), "which","were","also","and","has","our","have","having",
"self","old","caused","when","most","among","out","from","uses","high","may","after","than","m
ore","between","different","can","less","should","fromed","follows","feelings","times","basis","w
eeks","xia","using", "two","show","showed","seven","pabc", "mainly","meet","based","joint","ri
ght","standard","limb","left","divide","numb","dynasty","video","three","period","fitness","chang
e","literature","chinese","method","main","name","effect","analysis","halphysical","difficulty","pr
ocessmeasure","human","think","social","purpunodel","power","give","time","game","key","histo
ry","late","china","qing","Seath","major","first","follow","field","one","low","play","find","resuse
t","modern","model","top","way","take","lack","mode","ltrain","early","rate","present","forward",
"trend","japan","difficult","explore","motion","will","movement","mean","improve","use","need",
"study","event","promote","good","state","military","reform","provide","selection","review","spor
t","development","train","rhythmic","gymnast","value","gymnastic","new","paper group","contro
l","intervention","experimental train","group","culture","performance","people","carry","make","
hip","highly","add","get","set","last","cause","still","four","short","many","effort","year","form","
young","help","put","back","full","however","etc","pay","abstract","result","competitive","signifi
cantly","great","paper","significant","total") # 去除停用词
```
